# Rapidly liver-clearable rare-earth core–shell nanoprobe for dual-modal breast cancer imaging in the second near-infrared window

**DOI:** 10.1186/s12951-021-01112-y

**Published:** 2021-11-17

**Authors:** Zhuxin Wei, Guangxin Duan, Baoxing Huang, Shanshan Qiu, Dandan Zhou, Jianfeng Zeng, Jiabin Cui, Chunhong Hu, Ximing Wang, Ling Wen, Mingyuan Gao

**Affiliations:** 1 Department of Radiology, The First Affiliated Hospital of Soochow University, Institute of Medical Imaging, Soochow University, 188 Shizi Street, Suzhou, 215000 Jiangsu China; 2 Center for Molecular Imaging and Nuclear Medicine, State Key Laboratory of Radiation Medicine and Protection, School for Radiological and Interdisciplinary Sciences (RAD-X), Soochow University, Collaborative Innovation Center of Radiation Medicine of Jiangsu Higher Education Institutions, 199 Renai Road, Suzhou, 215123 Jiangsu China

**Keywords:** Rapid liver clearance, NIR-II region, Rare-earth core–shell nanoparticles, Dual-modal imaging

## Abstract

**Background:**

Fluorescence imaging as the beacon for optical navigation has wildly developed in preclinical studies due to its prominent advantages, including noninvasiveness and superior temporal resolution. However, the traditional optical methods based on ultraviolet (UV, 200–400 nm) and visible light (Vis, 400–650 nm) limited by their low penetration, signal-to-noise ratio, and high background auto-fluorescence interference. Therefore, the development of near-infrared-II (NIR-II 1000–1700 nm) nanoprobe attracted significant attentions toward in vivo imaging. Regrettably, most of the NIR-II fluorescence probes, especially for inorganic NPs, were hardly excreted from the reticuloendothelial system (RES), yielding the anonymous long-term circulatory safety issue.

**Results:**

Here, we develop a facile strategy for the fabrication of Nd^3+^-doped rare-earth core–shell nanoparticles (Nd-RENPs), NaGdF_4_:5%Nd@NaLuF_4_, with strong emission in the NIR-II window. What’s more, the Nd-RENPs could be quickly eliminated from the hepatobiliary pathway, reducing the potential risk with the long-term retention in the RES. Further, the Nd-RENPs are successfully utilized for NIR-II in vivo imaging and magnetic resonance imaging (MRI) contrast agents, enabling the precise detection of breast cancer.

**Conclusions:**

The rationally designed Nd-RENPs nanoprobes manifest rapid-clearance property revealing the potential application toward the noninvasive preoperative imaging of tumor lesions and real-time intra-operative supervision.

**Graphical abstract:**

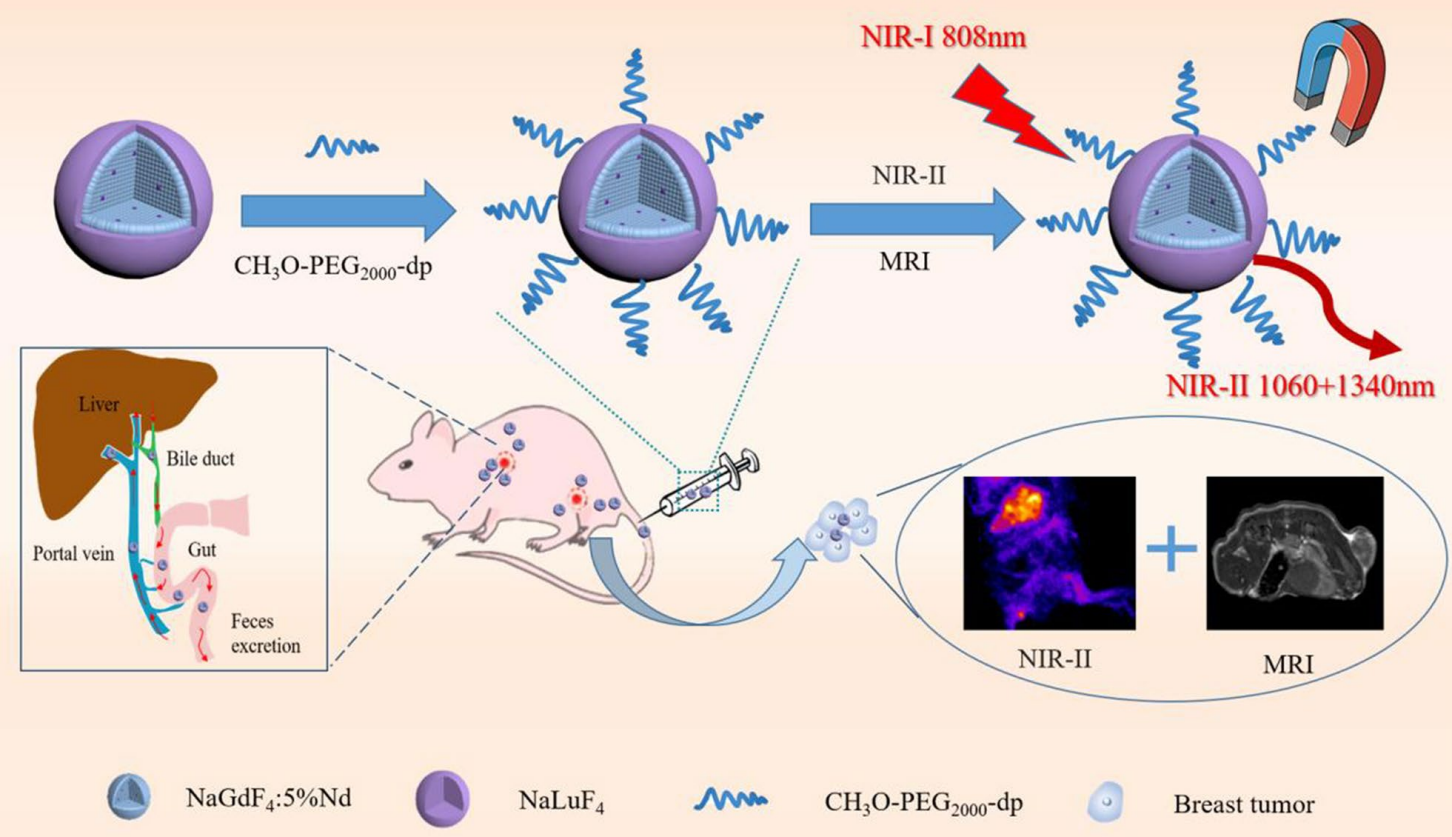

**Supplementary Information:**

The online version contains supplementary material available at 10.1186/s12951-021-01112-y.

## Background

Malignant tumor, one of the deadliest diseases in the world, threatens the health of human beings necessitating uninterrupted effort toward the accurate controlled diagnosis and therapeutic strategy of cancer, conversely, promoting the development of high precise, and high efficiency technology in the clinic. Recently, the optical method attracts significant attention due to its capability to offer noninvasive, high spatial resolution and the ability to provide physiological and pathological information for diverse biomedical applications [1–3]. Nevertheless, the traditional optical technology based on UV or Vis light prevents further development owing to its low absorption and scattering, reduced background auto-fluorescence interaction by the tissue [4–6]. Hence, it’s urgent to explore a new strategy that satisfied the future clinical demand.

NIR fluorescence probe with deep tissue penetration and high signal-to-noise ratio presents significant advantages toward clinical diagnostics and interventions. Up to now, the NIR fluorescence dye indocyanine green (ICG) has been approved by the US Food and Drug Administration (FDA) [7]. To this end, the commercially available ICG probe has been widely used for the tracing of lymph nodes in breast and gastric cancers [8, 9]. In order to meet the multilevel and diverse demands in clinic, it is essential to exploit the high resolution and precision in vivo imaging technology based on the NIR-II probe [10–12]. Now various NIR-II fluorescence probes, including rare earth-doped nanoparticles (RENPs) [13, 14], quantum dots semiconductor [15, 16], single-walled carbon nanotubes [17–19], and organic molecules [20, 21], have been widely developed in recent years. Among these probes, RENPs manifest special advantages, such as large Stokes shift, narrow and multi-peak emission profiles, and photostability, which make them potent for biomedical applications [22–24]. For instance, Ren et al. reported an Er-based lanthanide nanoparticle with strong NIR-II fluorescence, which enables the imaging-guided surgery of orthotopic glioma [25]. Moreover, Cheng et al. synthesized Nd^3+^-doped rare earth nanoparticles, which were appropriated for NIR-II and T2-weighted MRI dual imaging in an orthotopic hepatocellular carcinoma [26]. These findings manifest that RENPs have wide prospects for biological imaging.

Nevertheless, extensive studies revealed that RENPs often severely accumulated in the RES organs (liver and spleen) [27] resulting in potential toxicity due to the release of rare earth metal ions, which is a significant hindrance to the application of RENPs in vivo imaging [27–29]. Therefore, the development of a rapid clearance RENP probe from RES and utilized for accurate diagnosis of diseases is urgent, which requires further investigation.

**Fig. 1:**
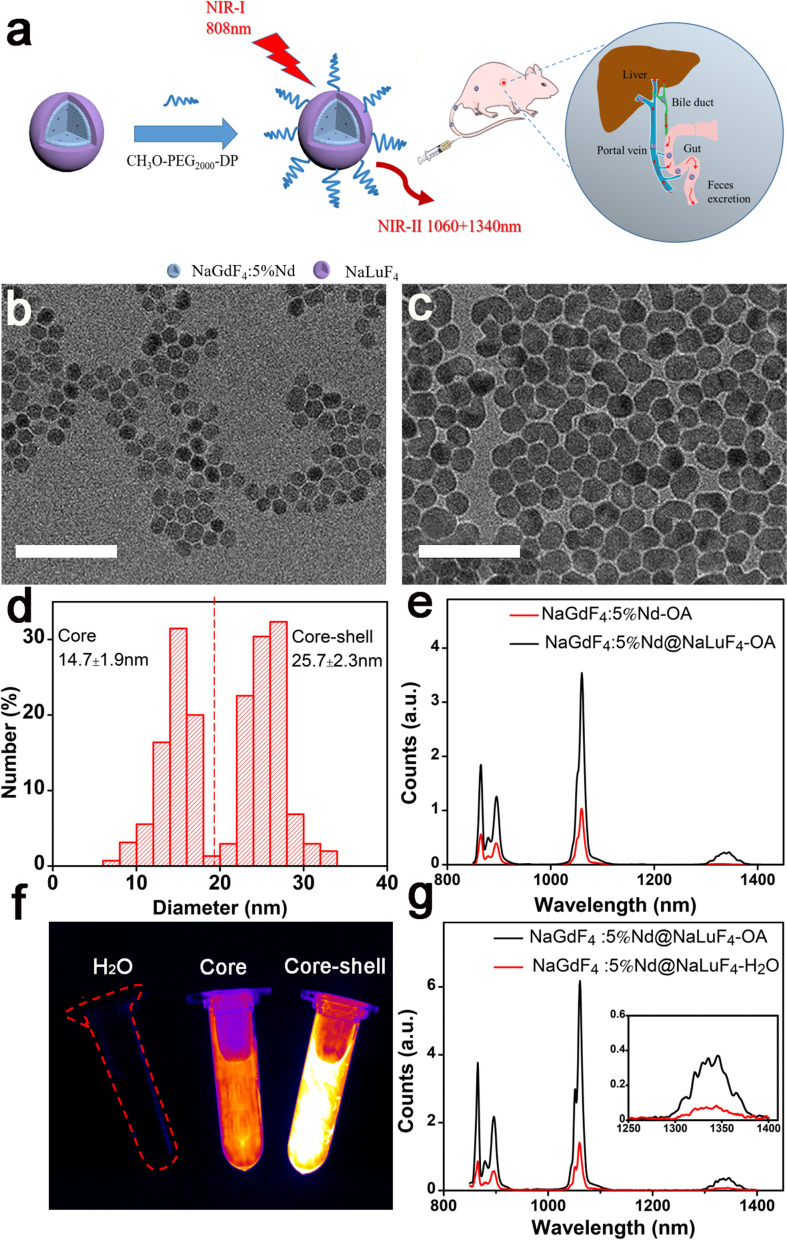
Characterizations of NaGdF_4_:5%Nd@NaLuF_4_ NPs. **a** Schematic illustration of Nd-RENP fabrication. **b** TEM image of NaGdF_4_:5%Nd cores (scale bar, 100 nm). **c** TEM image of NaGdF_4_:5%Nd@NaLuF_4_ core–shell NPs (scale bar, 100 nm). **d** Diameter distribution of core and core–shell RENPs. **e** Downconversion photoluminescence spectra of core RENPs (NaGdF_4_:5%Nd) and core–shell RENPs (NaGdF_4_:5%Nd@NaLuF_4_) under 808 nm excitation laser. **f** The fluorescence image of H_2_O, core, and core–shell RENPs was obtained using an 808 nm excitation laser. The fluorescence signals were collected via a 1000 nm long-pass filter. **g** Fluorescence emission spectra of OA-modified Nd-RENPs and PEGylated Nd-RENPs under 808 nm laser

Here, we report a facile strategy for the synthesis of Nd^3+^-doped RENPs with NIR-II fluorescence property and high signal-to-background ratio. After the surface modification by polyethylene glycol (PEG) consisting of a methoxy group at one end and a diphosphate group at the other end, the Nd-RENPs present good biocompatibility based on the in vitro and in vivo test, yielding the potential clinical applications. Interestingly, the Nd-RENPs probe could be excreted quickly from the RES system via the hepatobiliary pathway (half-life of 15.8 h in the liver) and rapid clearance from the blood circulation system (Fig. 1a). What’s more, the high resolution dual-modal imaging technology, including the NIR-II optical and MRI method, is utilized for sensitive detection of breast tumor with explicit boundary information. Overall, these findings revealed that the Nd-RENPs with the properties of good fluorescence performance and rapid clearance from the liver are potential probes for the real-time imaging of breast cancer in the NIR-II window.

## Materials and methods

### Materials and reagents

Gadolinium (III) chloride hexahydrate (GdCl_3_·6H_2_O, 99.9%), lutetium (III) chloride hexahydrate (LuCl_3_·6H_2_O, 99.99%), neodymium (III) chloride hexahydrate (NdCl_3_·6H_2_O, 99.9%), sodium fluoride (NaF, 99.99%), sodium hydroxide (NaOH, 96%), oleic acid (OA, 85%), and 1-octadecene (ODE, 90%) were purchased from Aladdin Co. Ltd. (Shanghai, China). Methyl thiazolyltetrazolium (MTT) was obtained from Sigma-Aldrich Co. Ltd. (Shanghai, China). Tetrahydrofuran (THF) of analytical grade was purchased from Shanghai Titan Scientific Co. Ltd. (Shanghai, China). ^99m^Tc was bought from Shanghai GMS Pharmaceutical Co., Ltd. (Shanghai, China). PEG (CH_3_O-PEG-DP, Mw: 2000) with diphosphate group at one end and methoxy group at the other end were customized products provided by Beijing Oneder Hightech Co. Ltd. (Beijing, China). All reagents were used as received without any purification.

### Synthesis of NaGdF_4_:5%Nd core nanoparticles

First, the ultra-small lanthanide fluoride nanocluster (NaLnF_4_ (Ln = Gd, Nd, Lu)) precursors were prepared in accordance with the liquid–solid-solution (LSS) strategy [30]. In a typical preparation, sodium hydroxide (1.2 g), deionized water (4 mL), 8 mL ethanol, and 20 mL OA were added in a 50 mL flask successively. After stirring for 10 min, 1 mL of gadolinium (III) chloride hexahydrate aqueous solution (0.5 mol/L) and 4 mL of sodium fluoride solution (0.5 mol/L) were added dropwise. The resultant solution was stirred at room temperature for 1 h until a yellowish solution formed. The solution was then precipitated with ethanol to collect NaGdF_4_ nanoclusters. Following washing with ethanol several times, the NaGdF_4_ nanoclusters were dispersed in cyclohexane (2 mL) for further use. The NaNdF_4_ and NaLuF_4_ nanoclusters were prepared similarly. The well-prepared NaGdF_4_ nanoclusters (0.25 mol/L) and NaNdF_4_ nanocluster solution (0.0125 mol/L) were later dissolved together in 2 mL cyclohexane. Subsequently, the NaGdF_4_/NaNdF_4_ solution was mixed with 6 mL OA and 10 mL ODE in a flask. The mixture was purged with nitrogen (N_2_) at 70 °C for 30 min to remove the cyclohexane thoroughly and then heated to 280 °C at a rate of approximately 10 °C/min and stirred for 30 min. After cooling to room temperature, the resultant nanoparticles were precipitated by ethanol and collected by centrifugation at 11,000 rpm for 5 min. Finally, precipitation was re-dispersed in cyclohexane for further experiments.

### Synthesis of NaGdF_4_:5%Nd@NaLuF_4_ core–shell nanoparticles

NaLuF_4_ shell was synthesized following a similar process to the NaGdF_4_:5%Nd preparation. The well-prepared NaGdF_4_:5%Nd nanoparticles, 0.5 mmol NaLuF_4_ nanocluster solution, and OA and ODE were added to a flask and reacted to fabricate OA-coated NaGdF_4_:5%Nd@NaLuF_4_ core–shell nanoparticles.

As a typical example, 100 mg of PEG-diphosphate ligand (DP-PEG_2000_) and 10 mg of OA-coated NaGdF_4_:5%Nd@NaLuF_4_ core–shell RENPs were mixed with 5 mL THF. The ligand exchange was then executed under the condition of stirring for 24 h at 40 °C. The PEG-coated particles were precipitated by cyclohexane, washed with cyclohexane thrice, and dried in a vacuum at room temperature for 4 h. The dried particles were dispersed in Milli-Q water and purified by ultrafiltration three times to remove free polymers. The resultant solution was finally dispersed in Milli-Q water for further use.

### Characterization

The size and morphology of the nanoparticles were captured with an FEI Tecnai G20 transmission electron microscope (TEM, FEI, USA) operating at an acceleration voltage of 200 kV. The hydrodynamic size was measured at 25 °C with Zetasizer Nano ZS90 (Malvern, UK) equipped with solid-state He–Ne laser (λ = 633 nm). The concentration of the rare-earth elements was measured by inductively coupled plasma-mass spectroscopy (ICP-MS). The down-conversion fluorescence spectra were recorded using FLS980 spectra (Edinburgh Instruments, UK) equipped with an 808 nm laser serving as the excitation source. The crystal structures of nanoparticles were characterized with the Shimadzu XRD-6000 X-ray diffractometer (XRD) equipped with Cu Ka1 radiation (λ = 0.15406 nm).

The quantum yields (QYs) of PEGylated Nd-RENPs in water were evaluated via the previously reported method that takes IR-26 dye as a standard (QYs = 0.5%) [31]. All fluorescence was collected by FLS980 spectra under excitation of 808 nm laser. The absorption spectra of Nd-RENPs and IR-26 were recorded with Lambda 25 ultraviolet–visible (UV–Vis) spectrometer (Perkin-Elmer, USA). The relative QYs of the RENPs-PEG were calculated in the following formula (1): $\Phi s\, = \,\Phi r \, \left( {{\text{F}}_{{\text{s}}} {\text{/ F}}_{{\text{r}}} } \right)\, \times \,\left( {{\text{A}}_{{\text{r}}} {\text{/ A}}_{{\text{s}}} } \right)\, \times \,\left( {{\text{n}}_{{\text{s}}}^{{2}} {\text{/ n}}_{{\text{r}}}^{{2}} } \right),$ where *Ф* represents QYs, and F_s_ and F_r_ respectively denote the integral areas of photoluminescence (PL) of Nd-RENPs and IR-26 under excitation of 808 nm laser [32, 33]. A_r_ and A_s_ are the NIR absorbance of the reference and samples at 808 nm, respectively, and n is the refractive index of the solvent (n_s_ = 1.33 for water, n_r_ = 1.44 for dichloroethane).

### Cytotoxicty of Nd-RENPs

The cytotoxicity of core–shell Nd-RENPs was assessed by MTT assays. Briefly, 4T1 cells were seeded into 96-well plates at a concentration of 6 × 10^3^ cell/well and cultured under the condition of 37 °C and 5% CO_2_ for 24 h. The cells were then incubated with media containing different concentrations of Nd-RENPs (0, 5, 10, 25, 50, 100, and 200 μg/mL), which were quantified with the concentration of gadolinium (Gd). The cells were added with MTT (5 mg/mL, 10 μL/well) after incubation for 24 h and incubated at 37 °C for 4 h. Thereafter, 100 μL of DMSO was added to each well. Following 15 min shaking, the 96-well plate was detected by EnSpire® Multimode Plate Reader (PerkinElmer, USA) at 490 nm. Cell viability was calculated in accordance with the formation: $\left( {{\text{A}}_{{{\text{sample}}}} - {\text{ A}}_{{{\text{blank}}}} /{\text{A}}_{{{\text{control}}}} - {\text{A}}_{{{\text{blank}}}} } \right)\, \times \,{1}00\%$ [34].

### Hemolysis test

First, 1 mL collected whole blood cells were added into 2 mL phosphate-buffered saline (PBS). Red blood cells (RBCs) were collected by centrifugation (500 g, 10 min). The above steps were repeated for 3 times to purify RBCs. Then, different concentrations of Nd-RENPs were added into freshly isolated RBCs. After incubation of 3 h under the condition of 37 °C, RBCs were centrifuged at 10,000 rpm for 5 min. Hemolysis capability was appraised by measuring the absorbance of supernatants at 540 nm. PBS treatment was acted as the negative control, and deionized water treatment was adopted as the positive control. The hemolysis ratio was calculated by the following formula: ${\text{hemolysis }}\% \, = \,\left( {{\text{sample absorbance}} - {\text{negative control absorbance}}} \right)/\left( {{\text{positive control absorbance }} - {\text{negative control absorbance}}} \right)\, \times \,{1}00\%$ [35].

### Animal tumor model construction

All animal experiments were executed in accordance with guidelines approved by the ethics committee of Soochow University (Soochow, China). Specific pathogen-free grade BALB/c female mice (4–5 weeks old) were selected and fed adaptively for one week before tumor-bearing. Tumor-bearing was conducted by subcutaneous inoculation of 5 × 10^6^ 4T1 cells into mice at the right flank region on the back of the mice.

### NIR-II fluorescence imaging

The nude mice were anesthetized with isoflurane, and then DP-PEG_2000_-modified Nd-RENPs were administered through intravenous injection (15 mg of Gd^3+^ per kilogram body weight). In vivo NIR-II fluorescence imaging was performed by the NIR-II Imaging System (Serious II 900–1700) under the excitation of 808 nm laser (45 mW/cm^2^). NIR-II photoluminescence images were obtained by using the long-pass filter of 1250 and 1000 nm. The tumor-to-background ratio (TBR) of two signal channels was calculated using the following equation: ${\text{TBR}}\, = \,\left[ {\left( {\text{mean fluorescence intensity of tumor}} \right) - \left( {\text{mean fluorescence intensity of background}} \right)} \right]/\left[ {\left( {\text{mean fluorescence intensity of normal tissue}} \right) - \left( {\text{mean fluorescence intensity of background}} \right)} \right]$. Additionally, liver-to-background ratio (LBR) was introduced as an evaluation index to evaluate the changing trend of liver signals in different periods (LBR = [(mean fluorescence intensity of liver)-(mean fluorescence intensity of background)]/[(mean fluorescence intensity of normal tissue)-(mean fluorescence intensity of background)]).

### Biodistribution of Nd-RENPs

The biodistribution of Nd-RENPs was analyzed by fluorescent quantitation and SPECT/CT imaging. The BALB/c mice (n = 3) were imaged under the NIR-II imaging system at different time points after intravenous administration for fluorescent quantitation. Subsequently, the mice were sacrificed at 1, 24, 48, and 72 h post-injection. The major organs and tissues, including heart, liver, spleen, lung, kidneys, small intestine, large intestine, feces, and stomach, were taken and imaged ex vivo under the NIR-II imaging system. Finally, fluorescence was quantitated to analyze the distribution of Nd-RENPs. Radioactive Technetium-99 m (^99m^Tc) was labeled on Nd-RENPs for SPECT/CT imaging through the chelating effect between the phosphate group and ^99m^Tc according to the reported method [36, 37]. The obtained ^99m^Tc-labeled nanoparticles were injected into BALB/c mice via tail vein with a 50 mCi/kg dose. The SPECT/CT images were then recorded at various time points by SPECT/CT scanner (MILabs, the Netherlands) [scan time: 10 min/frame; Field of view (FOV): 26 × 26 × 70 mm^3^; resolution: 0.6 mm]. The acquired SPECT/CT images were reconstructed by MILabs software and fused with PMOD software. Quantification was performed by selecting the desired organs using the quantification tool of PMOD software.

### Blood circulation behavior of Nd-RENPs

Three healthy BALB/c mice were intravenously injected with the Nd-RENPs at a dosage of 10 mg/kg. Blood samples were drawn from their eye sockets at different time points (5, 10, 15, 30, and 45 min; 1, 1.5, 2, 4, 8, 12, 24, and 48 h) after injection. The contents of Gd in the blood samples were measured by ICP-MS after digestion with the mixture of HNO_3_ and H_2_O_2_. The decay curve of Gd contents in the blood was fitted with an exponent quadratic model.

### In vivo MRI

MRI was performed through a 3-Tesla MRI scanner (MR Solution, UK) at 5, 15, 30, 60, 90, 120, and 240 min after Nd-RENP (15 mg/kg) administration. Multislice coronal T1-weighted images were obtained from the abdomen of each mouse by using a fast spin-echo sequence with fat saturation [repetition time (TR) / echo time (TE) = 850/11 ms, Flip angle (FA) = 90, FOV = 40]. The tumor contrast was assessed by ImageJ software after Nd-RENPs administration.

### Histological analysis

Major organs (heart, liver, spleen, lung, and kidney) of mice were harvested on day 7 after intravenous injection to assess the histological toxicity caused by Nd-RENPs. The histological toxicity was evaluated by optical microscope following placement in 10% neutral buffered formalin, routine processing into paraffin, sectioning into thin slices, and performing hematoxylin–eosin staining (H&E).

## Results and discussion

### Synthesis and characterization

Figure 1a displayed the schematic illustration of the NaGdF_4_:5%Nd@NaLuF_4_ NPs. Firstly, different NaLnF_4_ nanoclusters (Ln = Gd, Nd, Lu) were prepared in accordance with the literature method called LSS, which is based on general phase transfer and separation mechanism [30, 38]. NaGdF_4_, NaNdF_4_, and NaLuF_4_ nanoclusters were respectively employed as core, dopant, and shell to achieve Nd-RENPs through reaction. TEM images showed that the average diameter of the core RENPs (NaGdF_4_:5%Nd) and core–shell RENPs (NaGdF_4_:5%Nd@NaLuF_4_) were approximately 14.7 ± 1.9 and 25.7 ± 2.3 nm, respectively (Fig. 1b–d). Some Nd-RENPs were spherical, while the others were short-rod shaped. The XRD peaks of Nd-RENPs (Additional file 1: Fig. S1a and b) were displayed consistent with the standard card of NaGdF_4_ (JCPDS: 27-0699) and NaLuF_4_ (JCPDS: 27-0726), indicating the good crystallinity of core–shell Nd-RENPs without significant changes with the adding of Nd^3+^ dopants. Thereafter, the fluorescence property of NPs was analyzed by FLS980 spectra. As indicated from the fluorescence spectra in Fig. 1e, the core RENPs (NaGdF_4_:5%Nd) and core–shell RENPs (NaGdF_4_:5%Nd@NaLuF_4_) possessed two emission peaks (1060 and 1340 nm) in the NIR-II region under the excitation of 808 nm laser. The fluorescence intensity of core–shell Nd-RENPs at the same molar concentration increased 3.4 times at 1060 nm comparing with that of the core NPs. This phenomenon is attributed to the NaLuF_4_ shell coating, which can effectively decrease the non-radiative transition process of Nd^3+^ ions [39]. The result was also verified by fluorescence imaging. Figure 1f shows that unlike H_2_O without fluorescence, core RENPs and core–shell RENPs demonstrated strong fluorescence under the excitation of 808 nm laser. The core–shell RENPs exhibited stronger photoluminescence intensity compared with core RENPs, which should be facilitated for the in vivo bioimaging. However, the oleate ligands on the surfaces of the RENPs contributed to the reduced solubility of particles in water, which was not conducive to their biomedical applications. The functional PEG polymer was adopted to replace the oleate ligand to improve the water solubility and biocompatibility of Nd-RENPs. The PEGylated Nd-RENPs (Additional file 1: Fig. S2) displayed the similar morphology to that of unmodified RENPs. The fluorescence spectra result revealed that the fluorescence intensity at 1060 nm was decreased by nearly 4.2 times after surface modification (Fig. 1g), namely, the ligands exchange step could damage the passivation effect and expose more surface defect resulting in the quenching of the PL of the synthesized NPs. The relative photoluminescence QYs were quantified to further verify the photoluminescence intensity of Nd-RENPs. The result (Additional file 1: Fig. S3) showed that the relative QY of Nd-RENPs was 0.89%, which is higher than the similar nanoprobe reported in previous literature [22, 38, 40]. In addition, our results indicated that the tissue penetration depth in chicken breast tissue is up to 1 cm (Additional file 1: Fig. S4).

The dynamic light scattering (DLS) and fluorescence spectra of Nd-RENPs at different time points were analyzed to evaluate the physicochemical stability and photostability of the PEGylated Nd-RENPs, which were important for their applications. The results of DLS displayed that PEGylated core–shell Nd-RENPs possessed a single scattering peak located at 32.7 nm in water, PBS (pH = 7.4), 10% FBS, and the peak remained the same within three days (Fig. S5a, b, c). Additionally, the NIR-II fluorescence intensity did not decrease in three days (Additional file 1: Fig. S5d, e, f), suggesting the high photostability of the NIR-II Nd-RENPs in water PBS and FBS. Moreover, as demonstrated in Additional file 1: Fig. S6, the zeta potential of Nd-RENPs remained nearly unchanged in 72 h. Those results demonstrated the good physicochemical stability of the Nd-RENPs. Herein, the fluorescence property and stability indicate the potential application in bioimaging.

### Toxicity assays

**Fig. 2:**
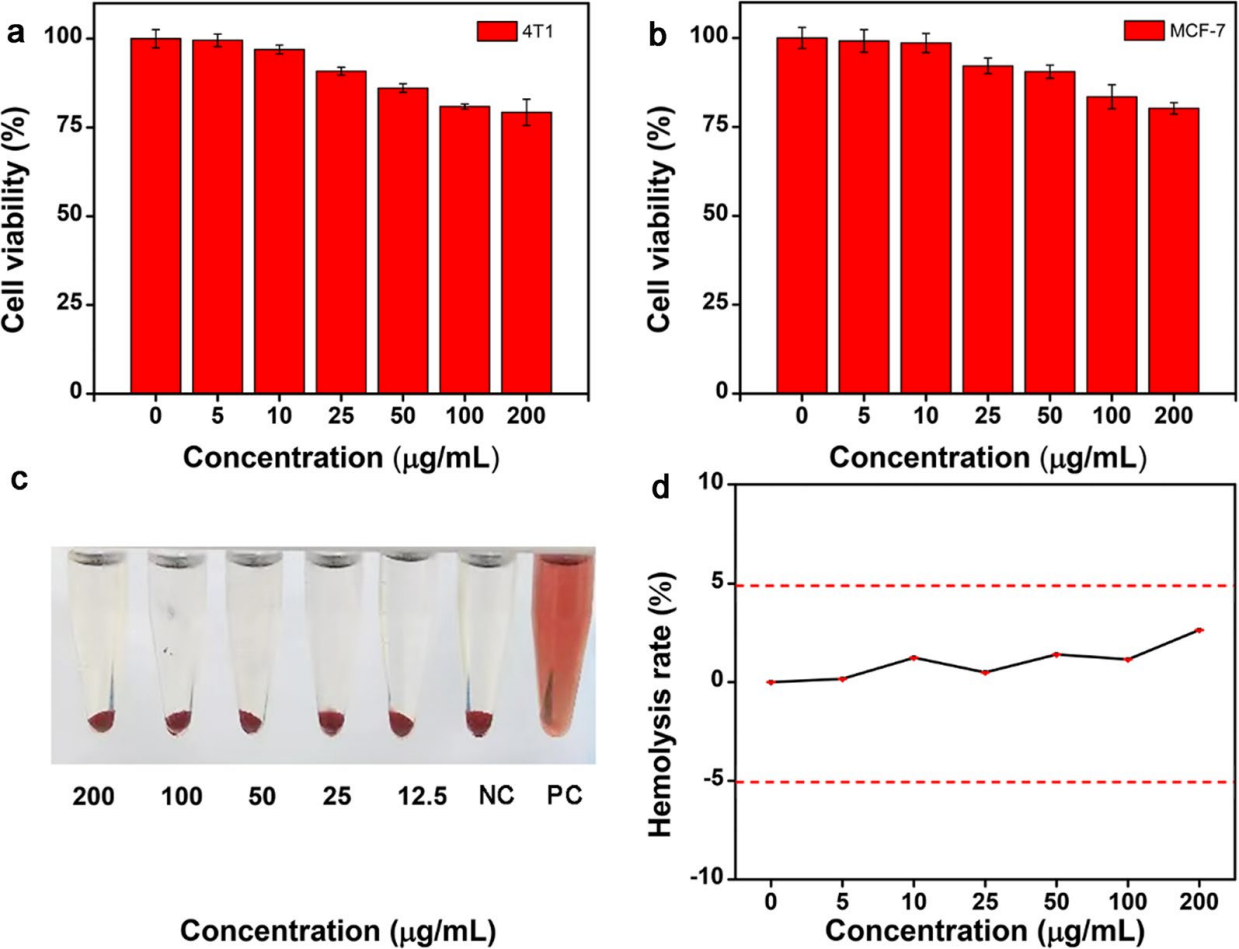
In vitro toxicity of Nd-RENPs. **a**, **b** Cytotoxicity caused by Nd-RENPs in 4T1 (**a**) and MCF-7 (**b**) cell lines was analyzed by MTT after incubation for 24 h. **c**, **d** Hemolysis activity of Nd-RENPs was appraised by color observation (**c**) and spectrophotometry (**d**)

Biocompatibility is essential for the application of imaging probes. The potential cytotoxicity of PEGylated core–shell Nd-RENPs was investigated in this study by MTT and hemolysis assays. Figure 2a and b showed that the cell viabilities (4T1 and MCF-7 cells) exceeded 80% after treatment with the Nd-RENPs for 24 h, even when the concentration of Gd^3+^ is as high as 200 µg/mL. Additionally, the hemolysis percentage of the RBCs was remarkably lower than 5% (limiting value) [41] after Nd-RENPs incubation, demonstrating the remarkable hemocompatibility of Nd-RENPs (Fig. 2c, d).

### NIR-II imaging of subcutaneous tumor

**Fig. 3:**
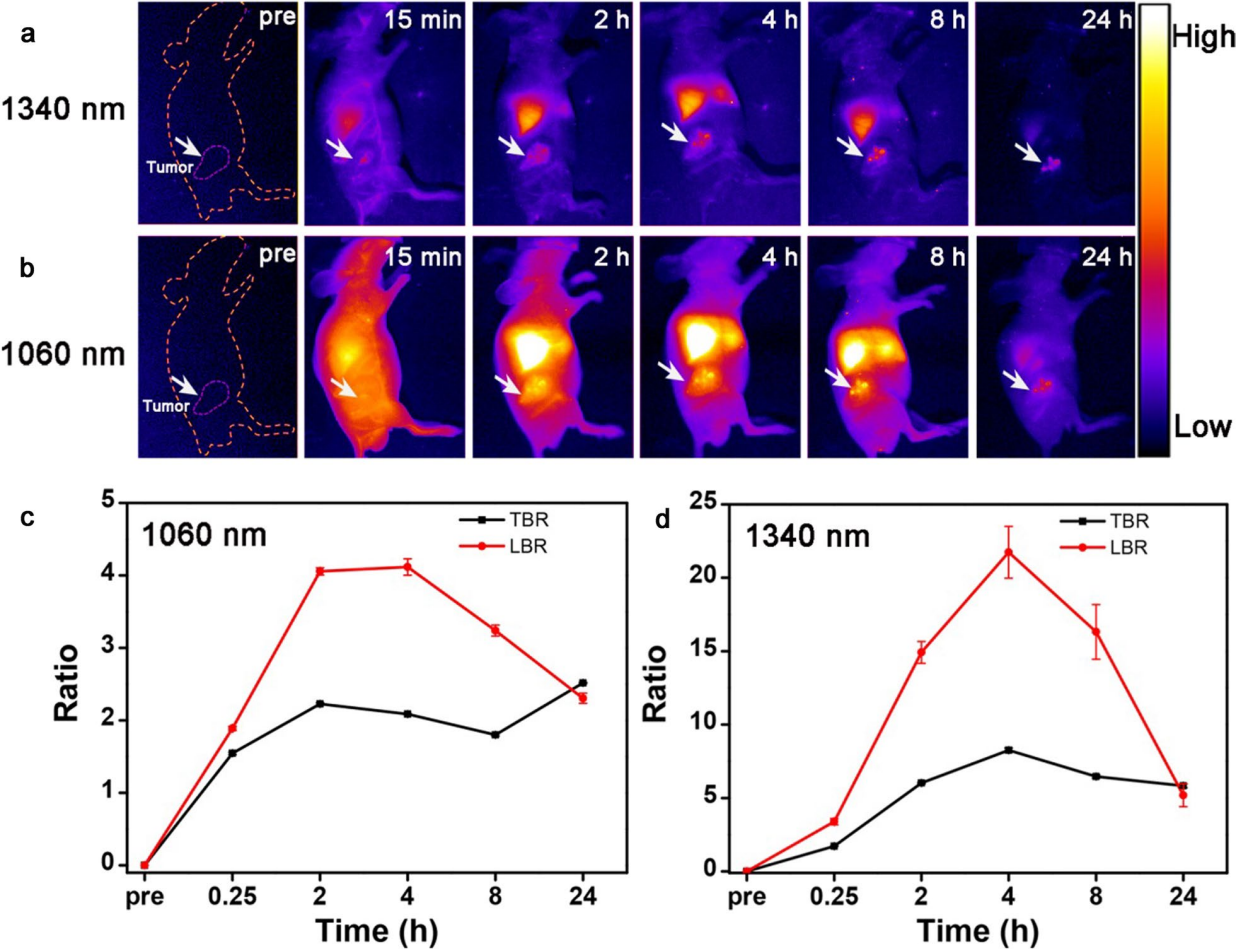
In vivo Imaging based on the Nd-RENPs in the NIR-II region. **a**, **b** NIR-II fluorescence images of 4T1-tumor-bearing mice at 1060 and 1340 nm after Nd-RENP administration. **c**, **d** TBR and LBR were calculated by the NIR-II fluorescence imaging of mice at 1060 and 1340 nm. TBR = [(mean fluorescence intensity of tumor)-(mean fluorescence intensity of background)]/[(mean fluorescence intensity of normal tissue)-(mean fluorescence intensity of background)]. LBR = [(mean fluorescence intensity of liver)-(mean fluorescence intensity of background)]/[(mean fluorescence intensity of normal tissue)-(mean fluorescence intensity of background)] (n = 3)

Optical imaging is a promising method for diagnosis because of the absence of radiation and high temporal resolution. Traditional fluorescence imaging in the visible light region is used in vitro due to its poor tissue penetration and signal to noise ratio (SNR). The NIR-II nanoprobes which are characterized by deep tissue penetration (up to several centimeter) [42], high imaging fidelity (even cover by 8 mm chicken breast tissue) [43] and weak tissue autofluorescence, are suitable for the diagnosis of in vivo imaging. Therefore, a simple subcutaneous model was constructed to evaluate the imaging performance of Nd-RENPs in the NIR-II region. Following the injection of Nd-RENPs (15 mg of Gd^3+^ per kilogram body weight) into the tumor-bearing mice via tail vein, NIR-II fluorescence images were acquired at different time points (15 min, 2, 4, 8, and 24 h). The multiplexed intravital NIR-II fluorescence imaging was conducted with the aid of different types of filters (long-pass filter of 1250 and 1000 nm) due to the fluorescence emission of Nd-RENPs at 1060 and 1340 nm. Figure 3a and b indicated the presence of fluorescence signal after 15 min post-injection. The NIR-II fluorescence signal increased in the first 4 h post-injection, and the signal reached the maximum at 4 h (Fig. 3c, d). Subsequently, the signal decreased significantly. Moreover, 1340 nm fluorescence has a lower background (TBR = 8.2) and higher resolution than that of 1060 nm (TBR = 2.5). Owing to the minimal autofluorescence, low scattering and absorbance of NIR-II emission at 1340 nm afford high SNR and deep tissue penetration depth comparing with that of fluorescence at 1060 nm, which facilitates the NIR-II optical imaging of tumor. Real-time visualization of tumors also opens the possibility of Nd-RENPs for accurate detection during surgery. Thus, Nd-RENPs are promising nanoprobes for the potential applications in surgical navigation of NIR-II imaging. Notably, the fluorescence signal in the liver rapidly decreased, and the LBR dropped from a peak of 21.7 at 4 h to 5.1 after administration for 24 h, suggesting the rapid clearance of Nd-RENPs from the liver. It is distinct from most of the NIR-II inorganic fluorophores that lingered in the liver [13, 44].

### In vivo pharmacokinetics of Nd-RENPs

**Fig. 4:**
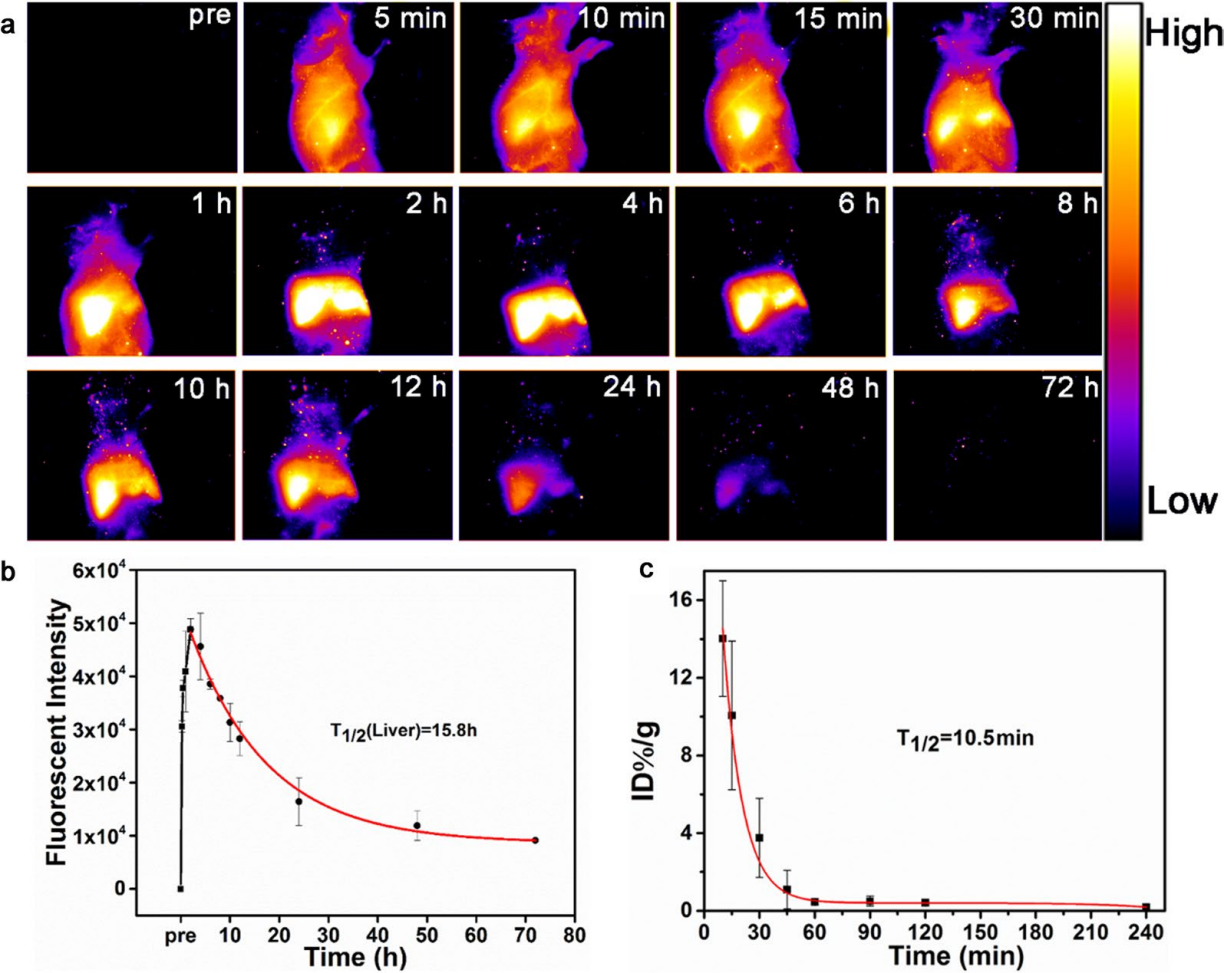
In vivo clearance behavior of Nd-RENPs. **a** NIR-II fluorescence images of a supine nude mouse after Nd-REN administration (Scale bar: 10 mm). **b** Quantitation analysis of NIR-II fluorescence in the liver (calculated half-life of the liver was 15.8 h) corresponding to Fig. 4a. **c** Blood circulation behavior of Nd-RENPs (n = 3)

Further experiments were performed to investigate the metabolism of the Nd-RENPs. Figure 4a demonstrated the gradual increase of the fluorescence signals in the liver after administration and peaking at 4 h. Subsequently, the signal in the liver began to decrease, and it was invisible after injection for 72 h, indicating the clearance of almost all particles from the liver. The in vivo half-life of Nd-RENPs in the liver was calculated as 15.8 h (Fig. 4b), which is significant short than the similar nanoprobes reported previously [13, 29, 44] and close to that reported by Yang and co-workers [45]. In addition, the pharmacokinetic results presented in Fig. 4c revealed that the blood half-life of nanoparticles is around 10.5 min, which contributed to rapid clearance in vivo. The high-magnification NIR-II imaging of hind-limb vessels was recorded after intravenous administration of core–shell Nd-RENPs due to the outstanding optical properties (Additional file 1: Fig. S8a). The Full Wave at Half Maximum (FWHM) was 804 µm (Additional file 1: Fig. S8b), which was measured by plotting the red line of vessel profiles in Additional file 1: Fig. S6a. The NIR-II signal intensity measured by the same vessel in the hind limb (Additional file 1: Fig. S8c) gradually declined during the 60 min post-injection, which illustrated the rapid blood clearance of Nd-RENPs.

**Fig. 5:**
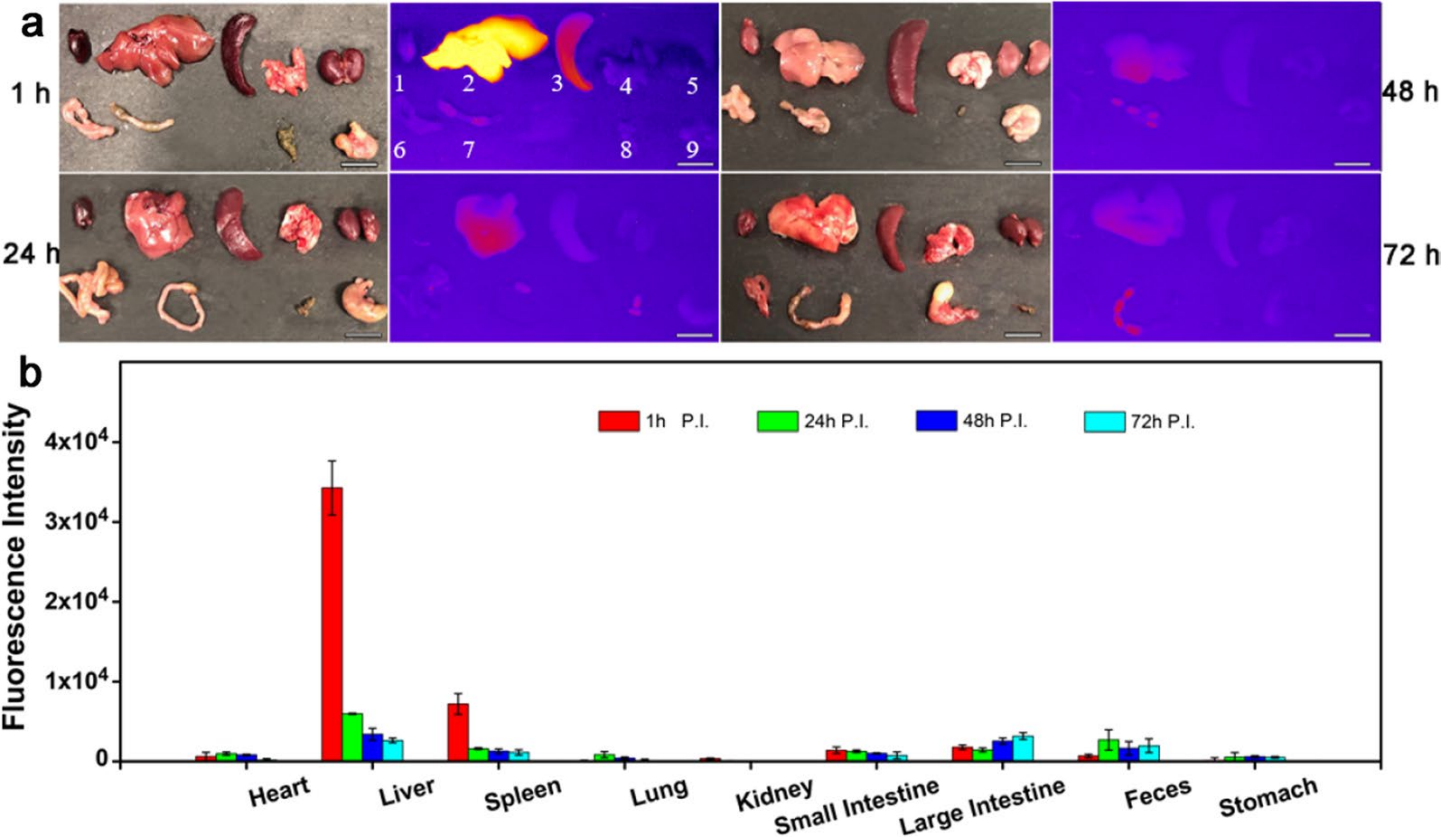
Biodistribution of Nd-RENPs. **a** Ex vivo biodistribution of vital organs was analyzed by NIR-II fluorescence intensity at 1, 24, 48, and 72 h after intravenous administration. **b** Quantitation analysis of NIR-II fluorescence intensity of vital organs. (1. Heart, 2. liver, 3. spleen, 4. lung, 5. kidney, 6. small intestine, 7. large intestine, and 9. stomach) and 8. feces at different time points. (Scale bar: 10 mm, n = 3)

The biodistribution of Nd-RENPs was conducted by fluorescence quantitation in the NIR-II region. It revealed that Nd-RENPs were mainly distributed in the liver and spleen after administration (Fig. 5a, b, respectively). The fluorescence signals in the liver and spleen significantly decreased with the prolongation of administration. The signal in the liver at 24 h post-injection was around five folds lower than that at 1 h and dropped over 90% at 72 h (Fig. 5a, b), which is also supported by the data of Gd3 + biodistribution analyzed by ICP-MS (Additional file 1: Fig. S7). Meanwhile, the fluorescence intensity in feces and large intestine contents was analyzed. Figure 5 shows that the signal was observed to be elevated at 24 h, which indicated that the Nd-RENPs might excrete through the pathway from bile to feces.

**Fig. 6:**
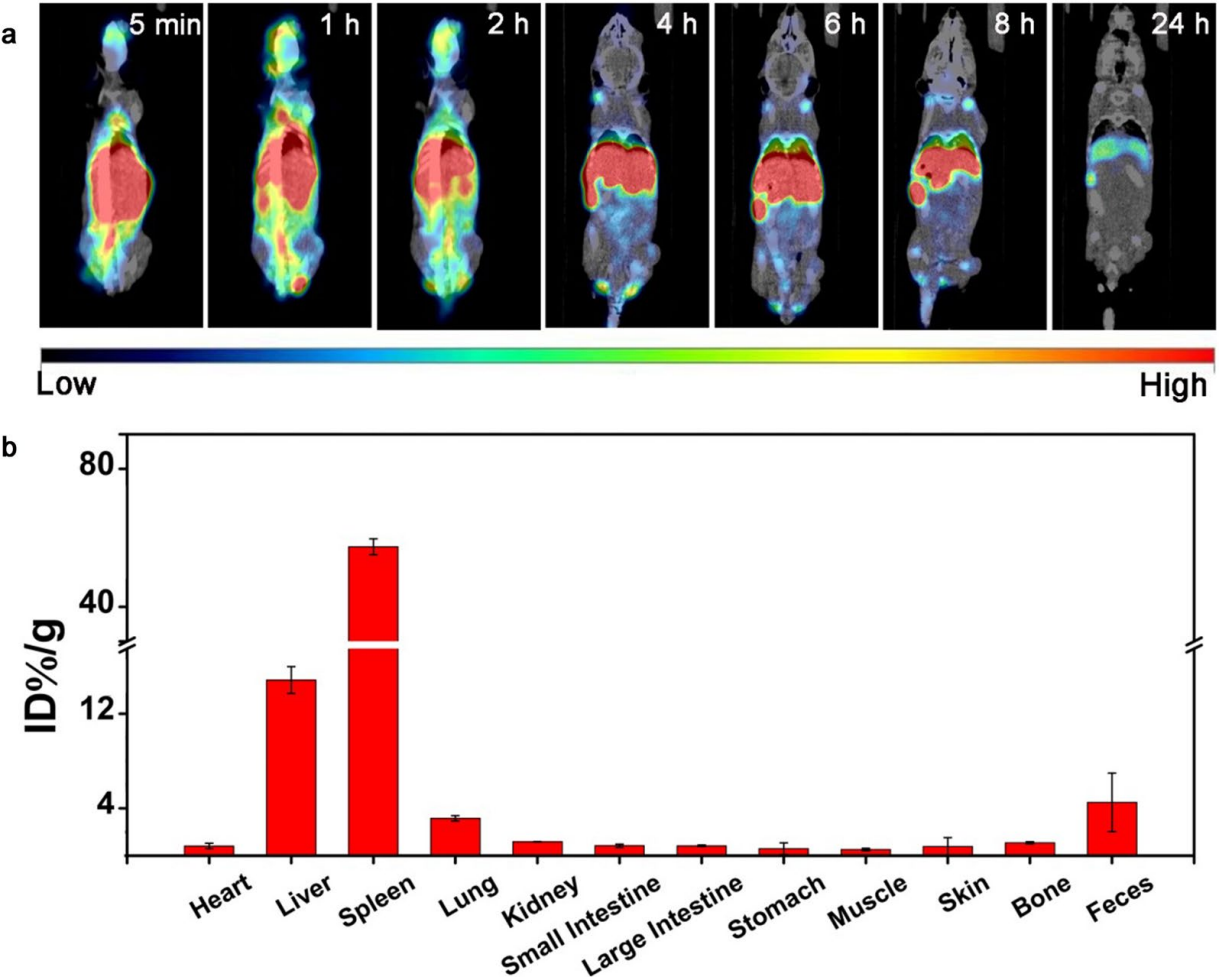
Biodistribution analysis by SPECT/CT imaging. **a** SPECT/CT images of mice after intravenous injection with the ^99m^Tc-labeled Nd-RENPs. **b** The quantified biodistribution of Nd-RENPs in different organs

Additionally, SPECT/CT was employed to further obtain comprehensive and real-time biodistribution information of Nd-RENPs, due to its outstanding temporal resolution [25, 36]. The results shown in Fig. 6a implicated that the Nd-RENPs were quickly Fed from the circulatory system and then taken up by the RES system organs (liver and spleen). Subsequently, intense signal in intestine was gradually captured, suggesting the excretion of Nd-RENPs through enterohepatic circulation from the liver to intestines. Moreover, the mice were sacrificed after injection for 24 h. The biodistribution of Nd-RENPs in vital organs was analyzed by a gamma counter. As indicated in Fig. 6b, the ID% g^−1^ value of the feces was significantly higher than that in the other organs (except liver and spleen), which also suggest the excretion pathway of Nd-RENPs through enterohepatic circulation. We speculated that the rapid excretion of Nd-RENPs in vivo might be related to the surface modification of PEG and the suitable short-rod shape of Nd-RENPs, which have been proven to facilitate nanomaterial escape from the capture of RES system and promote the excretion of nanoparticles through the hepatobiliary route [32, 35, 36].

### Magnetic resonance imaging of subcutaneous tumor

**Fig. 7:**
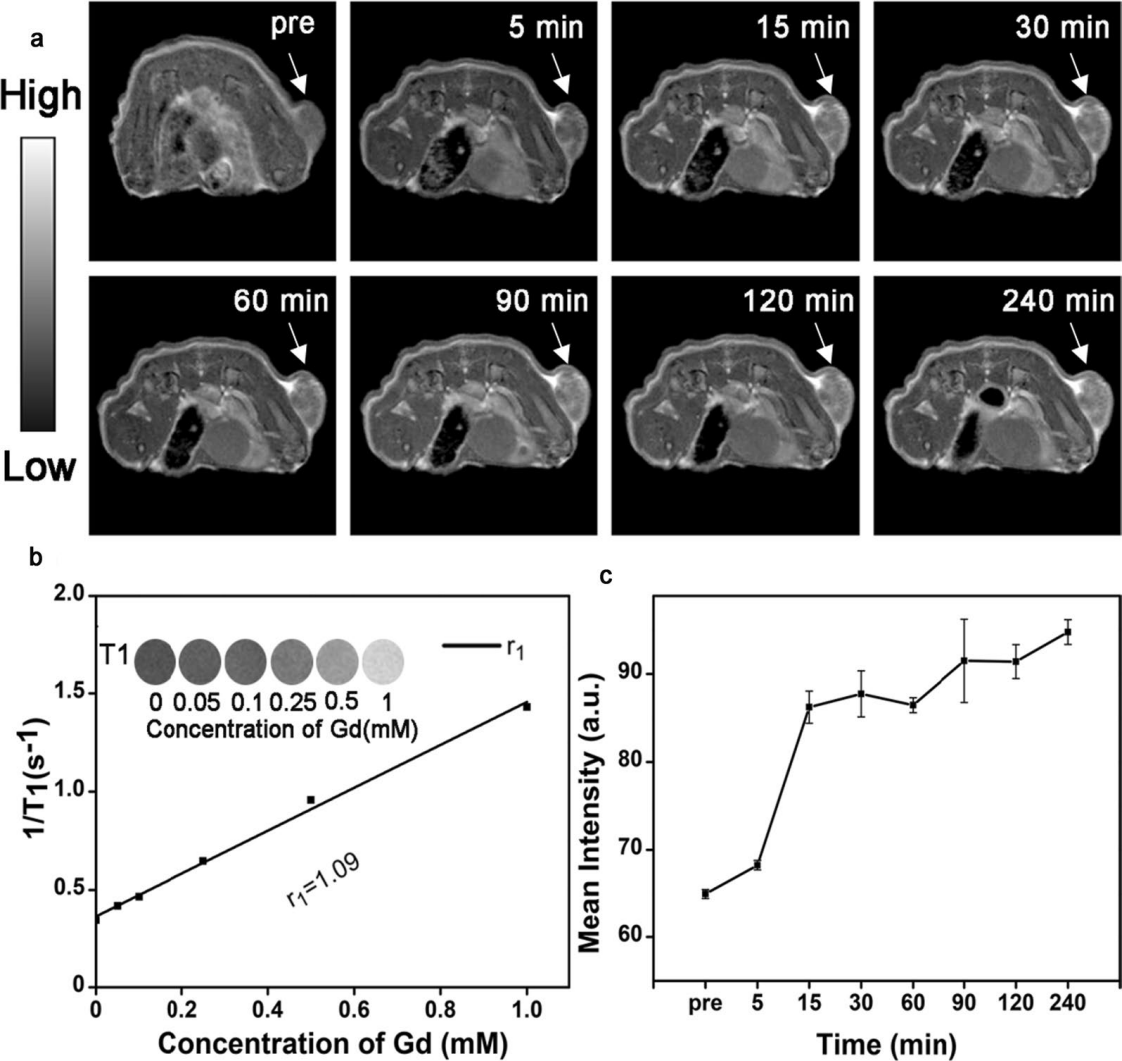
In vivo MRI of tumor-bearing mice. **a** T_1_-weighted MRI of breast tumor after injection with Nd-RENPs for 0, 5, 15, 30, 60, 90, 120, and 240 min. The tumor area was marked with arrow. **b** T_1_ relaxivity plot of the aqueous suspension of Nd-RENPs. **c** Mean intensity of MRI signals of tumors, n = 3

Although NIR-II imaging possesses superior temporal resolution, its low tissue resolution remains a problem. MRI, which is widely used in clinical diagnosis, has high tissue resolution. The integration of the two imaging technologies can effectively improve the diagnosis efficiency of the diseases. The design of NIR-II and MRI dual-functional probe is critical to the realization of the aforementioned integration. Rare-earth ions, such as Gd^3+^, Dy^3+^, and Ho^3+^, are potent agents used to relax the water protons for MRI because they have either a large number of unpaired electrons in the 4f orbitals and/or a large magnetic moment [46, 47]. In addition to the fluorescence performance in the NIR-II region, the core–shell Nd-RENPs exhibit interesting magnetism due to the existence of Gd^3+^. The longitudinal proton relaxation times (T1) relaxivity coefficient of the Gd-based Nd-RENPs was measured via a small MRI scanner under a 3 T magnetic field to investigate the MRI capability. Figure 7b shows that 1/T1 depended on the concentration of Gd^3+^, and the r_1_ value of Nd-RENPs was 1.09 mM^−1^ s^−1^, smaller than the rare earth nanoprobe we have reported previously [48], that may attributed to bigger size and less Gd^3+^ content of Nd-RENPs. T1-weighted MRI was performed on breast tumor-bearing mice to evaluate the dual-modal imaging capacity of Nd-RENPs in vivo. The results displayed that the MRI signals of subcutaneous tumors gradually increased and progressively moved toward the center of the lesion (Fig. 7a). The signal enhanced by 1.46-fold at 240 min compared with the previous one (Fig. 7c). Hence, as a potential MRI contrast agent, Nd-RENPs can provide complementary information for NIR-II imaging and improve the diagnosis effect of breast cancer.

### Biocompatibility of Nd-RENPs

**Fig. 8:**
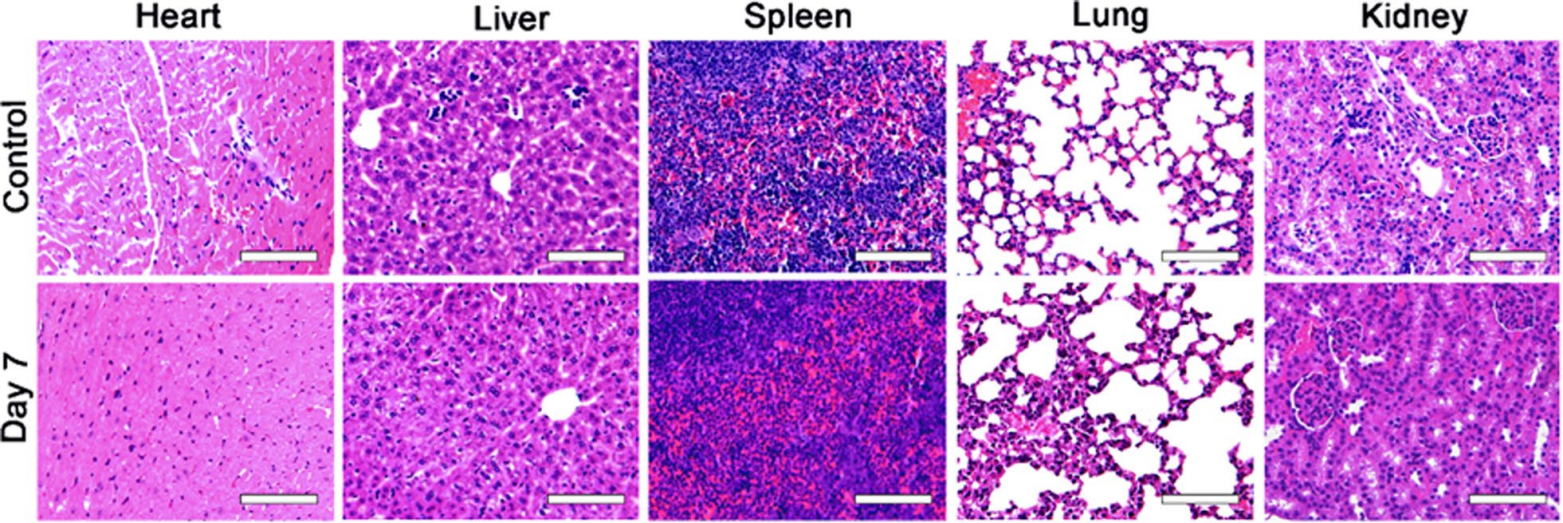
Biocompatibility evaluation by histological analysis of major organs (heart, liver, spleen, lung, and kidneys) at 7 d after intravenous administration (Scale bar: 100 μm)

Finally, H&E staining of major organs (heart, liver, kidney, spleen, and lung) was employed to further evaluate the biosafety of the Nd-RENPs. No clear pathological injury was observed from mice in the experimental groups after Nd-RENP administration for seven days compared with mice from the control group (Fig. 8). In addition, blood biochemical indices including alanine aminotransferase (ALT), aspartate aminotransferase (AST), creatinine (Cre) were analyzed, and none of these blood biomarkers were significantly altered compared with the control groups (Additional file 1: Fig. S9). These results revealed that the Nd-RENPs, as dual-imaging contrast agents, possess biocompatibility, which further illustrated their potential application in clinical diagnosis and imaging-guided surgery.

## Conclusions

A kind of excretable NIR-II rare-earth nanoparticles, NaGdF_4_:5%Nd@NaLuF_4_ core–shell Nd-RENPs, has been successfully synthesized in this study with high biocompatibility. The nanoparticles enabled optical-guided tumor detection without invasion and high spatial resolution sensing in vivo for intraoperative identification and navigation with the help of NIR-II imaging modality. Additionally, a comprehensive examination by several methods verified that most of the Nd-RENPs could be eliminated in vivo within 72 h via hepatic clearance route, which might be relative to the surface modification of Nd-RENPs and their short-rod shape which have been shown to be easily cleared from RES. Meanwhile, as multifunctional nanoprobes, Nd-RENPs could provide comprehensive MRI information pre-operatively. Moreover, in vitro and in vivo assays demonstrated the biocompatibility of Nd-RENPs. Consequently, this study reveals that the core–shell Nd-RENPs with the properties of suitable fluorescence and rapid metabolism have considerable value for clinical application in the accurate diagnosis of breast cancer.

## Supplementary Information

**Additional file1:** **Figure S1.** Powder X-ray diffraction (XRD) patterns for NaGdF_4_:5%Nd@NaLuF_4_ nanoparticles. **Figure S2.** TEM images of PEG-modified NaGdF_4_:5%Nd@NaLuF_4_ nanoparticles. **Figure S3.** Down-conversion luminescence spectra of NaGdF_4_:5%Nd@NaLuF_4_ nanoparticles and Dye IR-26. **Figure S4.** Comparison of the penetration and resolution of the Nd-RENPs at 1060 nm and 1340 nm. **Figure S5.** Stability analysis of core–shell Nd-RENPs. **Figure S6.** Zeta potential of the PEGylated Nd-RENPs. **Figure S7.** Biodistribution of Nd-RENPs was analyzed through ICP-MS. **Figure S8.** NIR-II imaging of the circulatory system. **Figure S9.** Blood biochemical indices were analyzed at 7 d after post-injection of Nd-RENPs. **Table S1.** The multimodal imaging of the lanthanum doped rare-earth NPs.

## Data Availability

The data and materials in the current study are available from the corresponding author on reasonable request.
