# Supplementary material for: Rapidly liver-clearable rare-earth core–shell nanoprobe for dual-modal breast cancer imaging in the second near-infrared window
Source: J Nanobiotechnology. 2021 Nov 17;19:369. doi: 10.1186/s12951-021-01112-y (PMC8600917; doi:10.1186/s12951-021-01112-y)
Supplement: Supplementary file 1 — Additional file1: Figure S1. Powder X-ray diffraction (XRD) patterns for NaGdF4:5%Nd@NaLuF4 nanoparticles. Figure S2. TEM images of PEG-modified NaGdF4:5%Nd@NaLuF4 nanoparticles. Figure S3. Down-conversion luminescence spectra of NaGdF4:5%Nd@NaLuF4 nanoparticles and Dye IR-26. Figure S4. Comparison of the penetration and resolution of the Nd-RENPs at 1060 nm and 1340 nm. Figure S5. Stability analysis of core–shell Nd-RENPs. Figure S6. Zeta potential of the PEGylated Nd-RENPs. Figure S7. Biodistribution of Nd-RENPs was analyzed through ICP-MS. Figure S8. NIR-II imaging of the circulatory system. Figure S9. Blood biochemical indices were analyzed at 7 d after post-injection of Nd-RENPs. Table S1. The multimodal imaging of the lanthanum doped rare-earth NPs. [file 12951_2021_1112_MOESM1_ESM.docx]

**Supporting Information for**

**Rapidly Liver-clearable Rare-earth Core-shell Nanoprobe for Dual-modal Breast Cancer Imaging in the Second Near-infrared Window**

Zhuxin Wei^1†^, Guangxin Duan^2†^, Baoxing Huang^2^, Shanshan Qiu^2^, Dandan Zhou^2^, Jianfeng Zeng^2^, Jiabin Cui^2^, Chunhong Hu^1^, Ximing Wang^1^*, Ling Wen^1^*, Mingyuan Gao^2^

^*^ Correspondence: wenling2011@qq.com, wangximing1998@163.com

^†^ Zhuxin Wei and Guangxin Duan contributed equally to the work

^1^ Department of Radiology, the First Affiliated Hospital of Soochow University, Institute of Medical Imaging, Soochow University, 188 Shizi Street, Suzhou, Jiangsu 215000, China

^2^ State Key Laboratory of Radiation Medicine and Protection, School for Radiological and Interdisciplinary Sciences (RAD-X), Collaborative Innovation Center of Radiation Medicine of Jiangsu Higher Education Institutions, Soochow University, 199 Renai Road, Suzhou, Jiangsu 215123, China

**
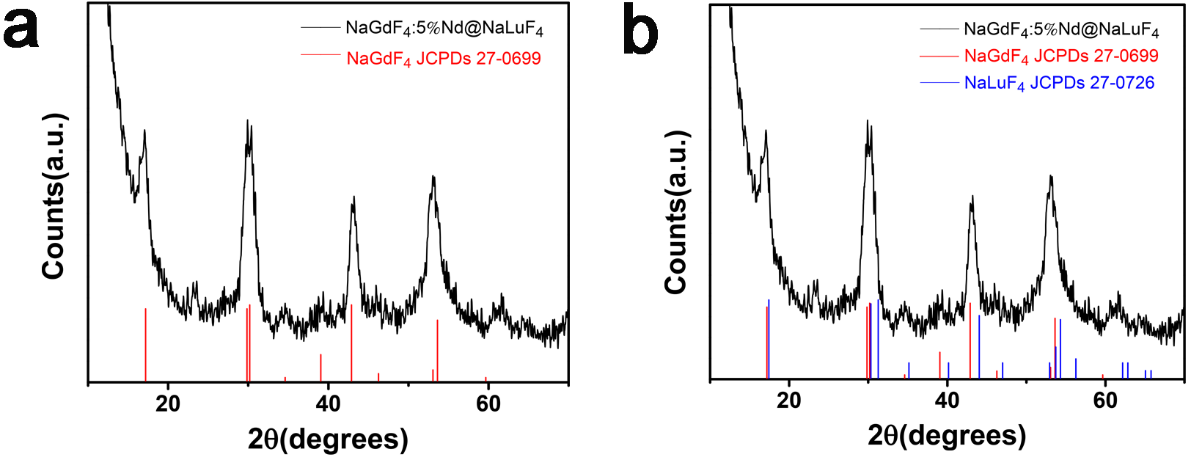
**

**Figure S1.** Powder X-ray diffraction (XRD) patterns for NaGdF_4_:5%Nd@NaLuF_4_ nanoparticles. a) JCPDS standard card No.27-0699 for hexagonal NaGdF4 crystal and b) No.27-0726 for hexagonal NaLuF_4_ crystal. XRD patterns were measured on a Shimadzu XRD-6000 X-ray diffractometer equipped with Cu Kα1 radiation (λ = 0.15406 nm).

**

**

**Figure S2.** TEM images of PEG-modified NaGdF_4_:5%Nd@NaLuF_4_ nanoparticles. (scar bar, 50 nm).


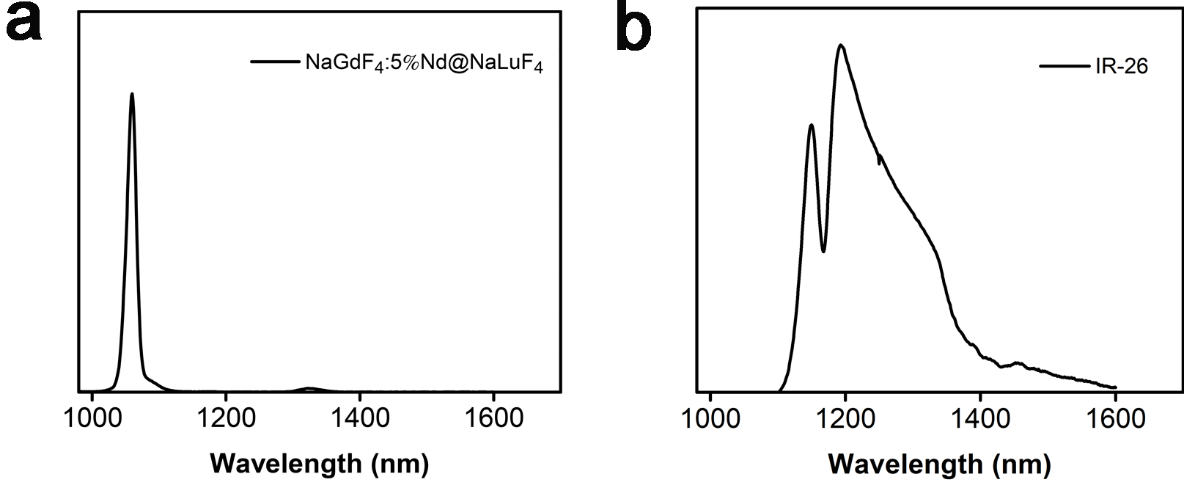


**Figure S3.** Down-conversion luminescence spectra of NaGdF_4_:5%Nd@NaLuF_4_ nanoparticles and Dye IR-26. a) Down-conversion luminescence spectra of NaGdF_4_:5%Nd@NaLuF_4_ dispersed in water (excitation wavelength is 808 nm). b) Down-conversion luminescence spectrum of Dye IR-26 dispersed in dichloroethane under 808 nm excitation.

*
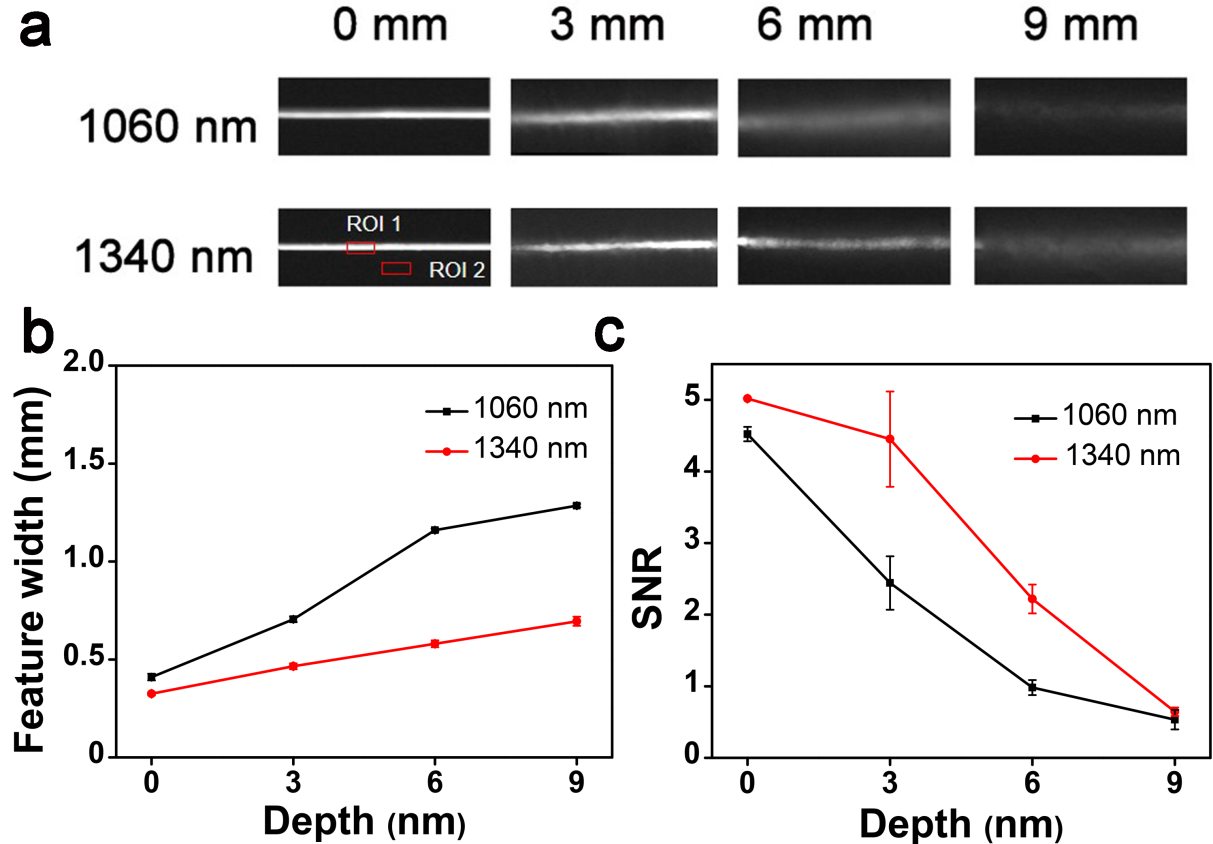
*

Fig. S4. Comparison of the penetration and resolution of the Nd-RENPs at 1060 nm and 1340 nm. a) The NIR-II images of the capillary glass tubes at 1060 nm and 1340 nm, when the tubes are covered with different thickness (0, 3, 6, 9 mm) of chicken breast tissues. b) The feature width of the NIR-II fluorescence signals in capillary glass tubes with variety depth of tissues. c) The SNR of the Nd-RENPs at 1060 nm and 1340 nm in different depth of tissues.


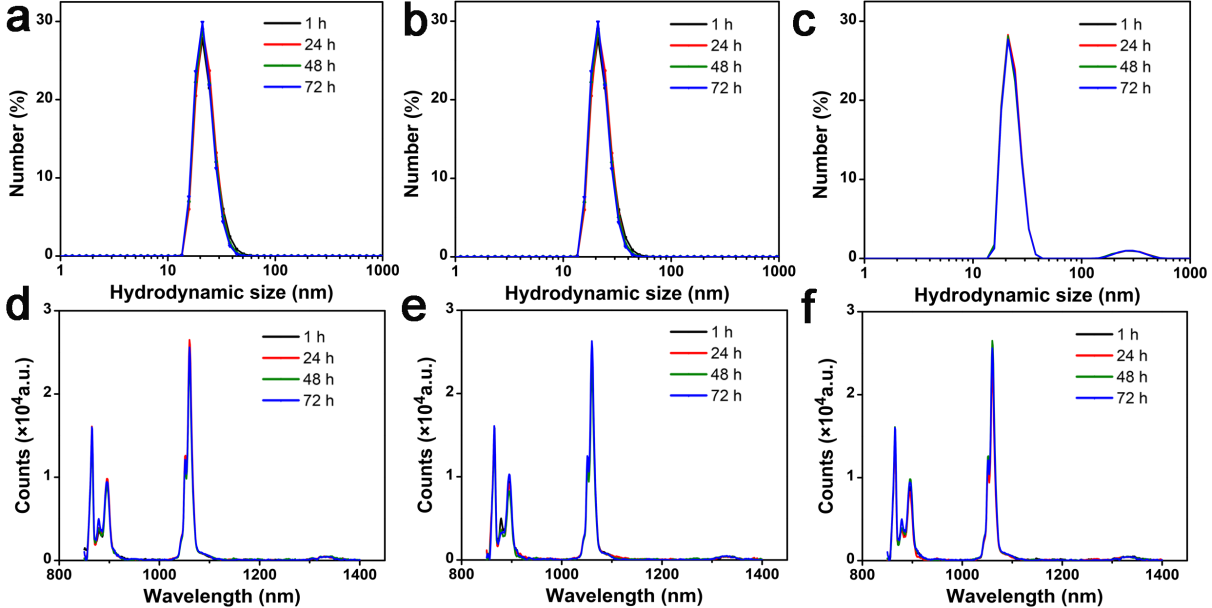


**Figure S5.** Stability of core-shell Nd-RENPs. a, b, c) Hydrodynamic size of nanoparticles in H_2_O, PBS and 10% FBS. d, e, f) Photostability of Nd-RENPs in H_2_O, PBS and 10% FBS.


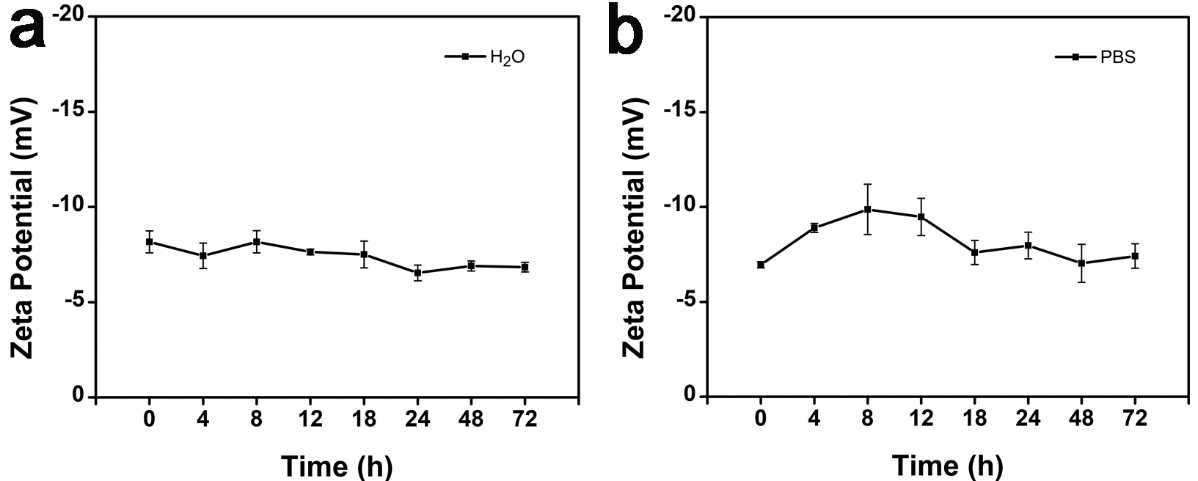


**Figure S6.** Zeta potential of the PEGylated Nd-RENPs in a) water and in b) PBS (n=3).

*
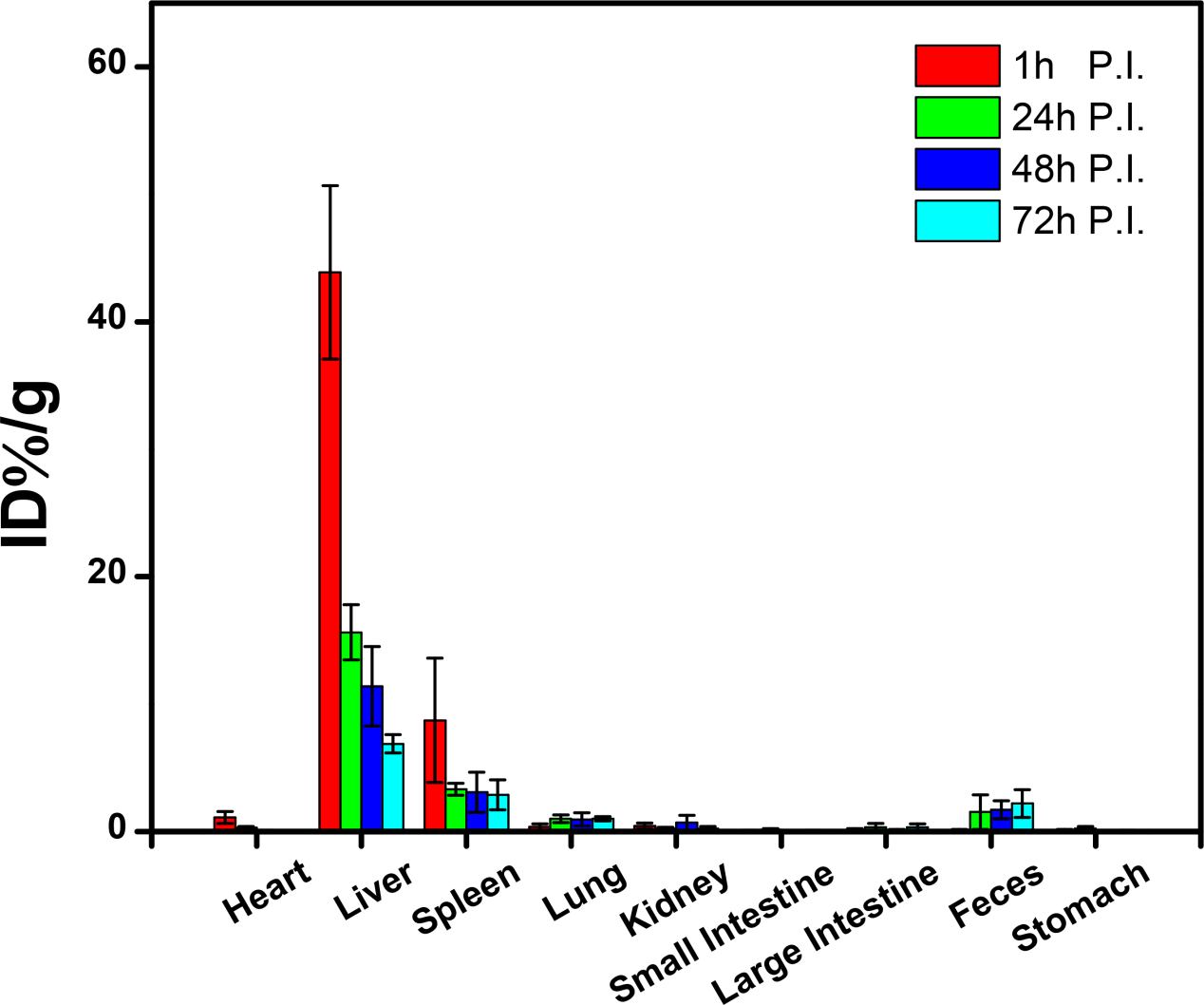
*

**Figure S7.** Biodistribution of Nd-RENPs was analyzed through measuring the contents of Gd^3+^ in major organs with ICP-MS (n=3).


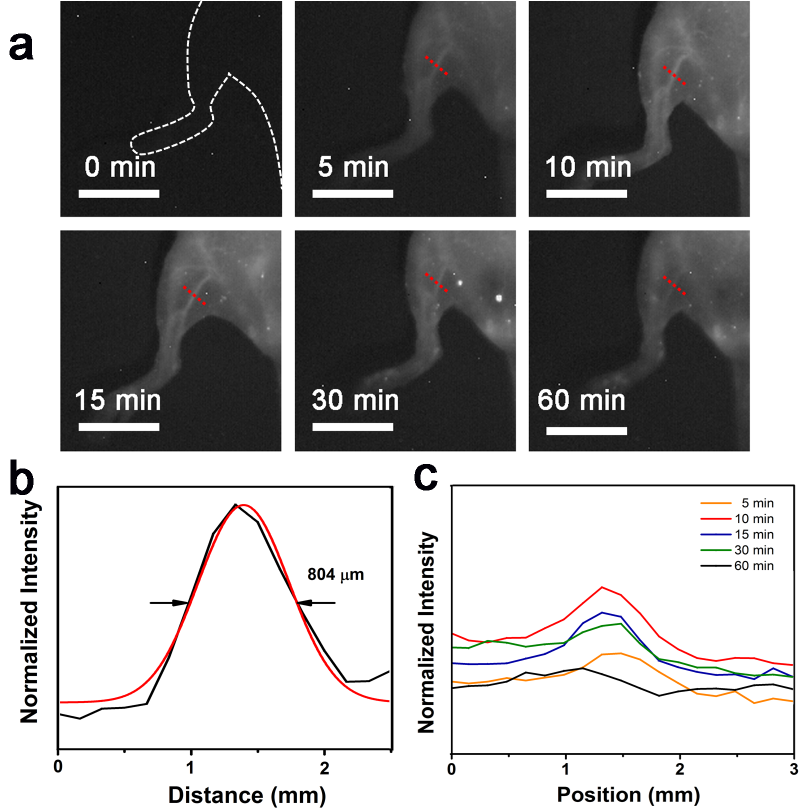


**Figure S8.** NIR-II imaging of the circulatory system. a) NIR-II imaging of femoral artery for 30 min after intravenous administration of core-shell NPs. b) Hind limb vascular and analysis of the vessel FWHM width; red lines in panel. c) Normalized NIR-II intensity of femoral artery during period of panels (a).


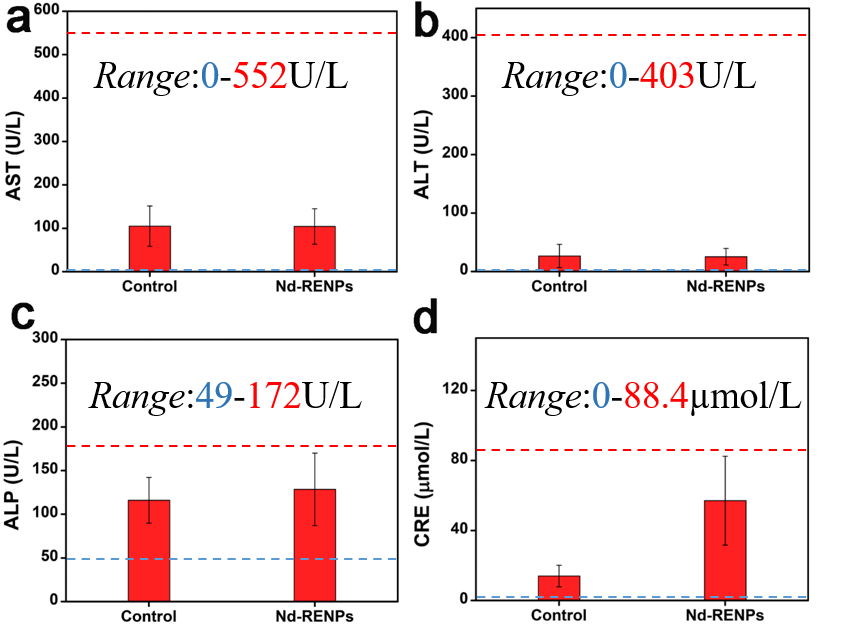


**Figure S9.** Blood biochemical indices including a) AST, b) ALT, c) ALP, and d) Cre were analyzed at 7 d after post-injection of Nd-RENPs (n=3).

Table S1. The multimodal imaging of the lanthanum doped rare-earth NPs.

| Samples | Multimodal imaging | References |
| --- | --- | --- |
| NaGdF_4_:5%Nd@NaLuF_4_  NaGd(WO4)2:Nd NPs  NaGdF_4_: Nd5 %@NaGdF_4_@Lips  NaYF_4_:Nd^3+^@NaLuF_4_  NaYF_4_:Yb^3+^, Tm^3+^, Co^2+^  NaYF4:Yb^3+^, Er^3+^@NaYbF_4_@NaYF_4_:Nd^3+^  FA-PEI-NaGdF_4_:Eu  PEG-^111^In-YPO_4_ | **NIR II and MRI**  NIR II, CT and MRI  NIR II/MRI  NIR II/CT  NIR I/MRI  NIR-II/PAI  NIR I/MRI  NIR-II/SPECT/CT | **Our work**  *Nano Research, 2021, 14(7): 2160–2170,*  *Nanoscale, 2020, 12(21): 11510-11517*  *ACS Appl. Mater. Interfaces,2017,9(32):* *26674-26683*  *Biomaterials, 2014,35(33): 9167-9176*  *Biosensors and Bioelectronics, 2020,151:112000*  *Talanta, 2017,165:161-166*  *Journal of Photopolymer Science and Technology,2016,29(4):525-532* |
